# Early intubation and patient-centered outcomes in septic shock: a secondary analysis of a prospective multicenter study

**DOI:** 10.1186/s13054-022-04029-6

**Published:** 2022-06-07

**Authors:** Ricard Mellado-Artigas, Carlos Ferrando, Frédéric Martino, Agathe Delbove, Bruno L. Ferreyro, Cedric Darreau, Sophie Jacquier, Laurent Brochard, Nicolas Lerolle

**Affiliations:** 1 Surgical Intensive Care Unit, Department of Anaesthesiology, Hospital Clínic, Institut D’investigació August Pi i Sunyer, Villarroel 170, 08025 Barcelona, Spain; 2 CIBER de Enfermedades Respiratorias, Instituto de Salud Carlos III, Madrid, Spain; 3 Medical and Surgical Intensive Care Unit, Guadeloupe University Hospital, Les Abymes, Guadeloupe France; 4 Medical and Surgical Intensive Care Unit, Centre Hospitalier Bretagne Atlantique, Vannes, France; 5 Department of Medicine, Sinai Health System and University Health Network, Toronto, Canada; 6 Interdepartmental Division of Critical Care Medicine, University of Toronto, Toronto, Canada; 7 Medical and Surgical Intensive Care Unit, Le Mans Hospital, Le Mans, France; 8 Medical Intensive Care Unit, Tours University Hospital, Tours, France; 9 Keenan Research Centre for Biomedical Science at the Li Ka Shing Knowledge Institute, St Michael’s Hospital, Toronto, ON Canada; 10 Medical Intensive Care Unit, Angers University Hospital and Angers Faculty for Health Sciences, Angers, France

**Keywords:** Septic shock, Outcomes, Tracheal intubation, Mechanical ventilation

## Abstract

**Purpose:**

Despite the benefits of mechanical ventilation, its use in critically ill patients is associated with complications and had led to the growth of noninvasive techniques. We assessed the effect of early intubation (first 8 h after vasopressor start) in septic shock patients, as compared to non-early intubated subjects (unexposed), regarding in-hospital mortality, intensive care and hospital length of stay.

**Methods:**

This study involves secondary analysis of a multicenter prospective study. To adjust for baseline differences in potential confounders, propensity score matching was carried out. In-hospital mortality was analyzed in a time-to-event fashion, while length of stay was assessed as a median difference using bootstrapping.

**Results:**

A total of 735 patients (137 intubated in the first 8 h) were evaluated. Propensity score matching identified 78 pairs with similar severity and characteristics on admission. Intubation was used in all patients in the early intubation group and in 27 (35%) subjects beyond 8 h in the unexposed group. Mortality occurred in 35 (45%) and in 26 (33%) subjects in the early intubation and unexposed groups (hazard ratio 1.44 95% CI 0.86–2.39, *p* = 0.16). ICU and hospital length of stay were not different among groups [9 vs. 5 (95% CI 1 to 7) and 14 vs. 16 (95% CI − 7 to 8) days]. All sensitivity analyses confirmed the robustness of our findings.

**Conclusions:**

An early approach to invasive mechanical ventilation did not improve outcomes in this matched cohort of patients. The limited number of patients included in these analyses out the total number included in the study may limit generalizability of these findings.

*Trial registration* NCT02780466. Registered on May 19, 2016.

**Supplementary Information:**

The online version contains supplementary material available at 10.1186/s13054-022-04029-6.

## Introduction

Invasive mechanical ventilation is a life-saving procedure and a key component of intensive care medicine. Despite its tremendous benefits, its use is associated with potential complications such as prolonged stay, acquired weakness, delirium and secondary infections [1–3]. For these reasons, there has been a growing interest to study the effectiveness of noninvasive strategies for providing respiratory support. So far, these modalities have been compared between them to study the benefits in reducing patients’ use of intubation and improving outcomes [4, 5].

Studies have described that failure to noninvasive support carries a poor prognosis in acute respiratory failure [6–10], suggesting that spontaneous ventilation can be injurious in situations where lungs have been primed for injury and strong breathing efforts take place [11]. Additionally, sedation and invasive ventilation are known to decrease metabolic needs, which could be desirable when tissue oxygen availability is low such as in shock states. Notwithstanding these potential benefits, there is high variability among clinicians regarding the decision to institute mechanical ventilation [12, 13].

Septic shock is one of the most common conditions that intensivists face every day, while heterogeneous, the general management focus on aggressive infection control and hemodynamic resuscitation with fluids and drugs. Mechanical ventilation is often used in this population, but guidelines do not offer clear advice about the optimal time to start this support. Besides the common downsides associated with mechanical ventilation, hemodynamic impairment after institution of positive pressure ventilation is of particular concern in this syndrome.

In the present study, we assessed the effect of intubation in septic shock patients in the first 8 h after vasopressor start (early intubation), as compared to non-early intubated controls on in-hospital mortality, intensive care and hospital length of stay in survivors and non-respiratory organ dysfunction at 24 h.

## Methods

This study represents a secondary analysis of a multicenter prospective registry (the INTUBATIC study) which assessed the timing and predictors of intubation in patients with septic shock [12].

### Patient population

The INTUBATIC study was a prospective, multicenter observational study conducted in 32 intensive care units (ICU) (thirty-one in France and a one in Spain) from May 2016 to October 2017 (NCT02780466) [12]. Patients aged 18 years old and above were eligible if admitted in a participating ICU for septic shock. Patients could be included if vasopressor was introduced in the 24 h preceding ICU admission. Patients were not eligible if vasopressor infusion was started after endotracheal intubation and mechanical ventilation. Patient, or a proxy if the patient was deemed unable to consent, received oral and written information about the study, and oral consent was obtained before inclusion. When a proxy gave the initial consent, patient’s consent was further obtained whenever possible. This study was approved by the Angers University Hospital ethics committee for all participating French hospitals (n° 2015/96).

For the present study, to assess the effect of early intubation on patients’ outcome, we excluded subjects that were intubated for surgery or any other invasive procedures. A preliminary communication has been submitted to the annual European Society of Intensive Care Medicine (ESICM) conference 2021.

### Study exposure and outcomes

In the current analysis, the main exposure of interest was the receipt of invasive mechanical ventilation in the first eight hours after vasopressor start in the ICU (early intubation or treatment group). In every case, the decision to intubate was left at the discretion of the treating physicians. The primary outcome of interest was in-hospital mortality. Secondary outcomes included ICU and hospital length of stay, up to 60 days from admission. Additionally, we studied the effect of early intubation on organ dysfunction as measured by non-respiratory, non-neurologic sequential organ assessment failure (SOFA) score at 24 h. Finally, an assessment of early intubation on fluid balance and the use of renal replacement at 24 h was also carried out. Number and percentage of patients with treatment limitation orders and median time to their onset were also assessed.

### Statistical analysis

Patient demographics, comorbidities, most abnormal vital signs and laboratory markers during the first eight hours after vasopressor infusion onset (and before intubation in those undergoing early mechanical ventilation) were compared between treatment groups in the study population. Time to intubation was calculated as the difference in hours from vasopressor start to intubation. Continuous variables were expressed as mean (standard deviation) or median (interquartile range, IQR). Categorical variables were presented as counts (percentages). To account for potential measured confounding on the effect of early intubation on study outcomes, we used multivariable logistic regression model to create a propensity score for receiving intubation in the first 8 h after vasopressor start. The criteria to include variables in these models were based on those potentially affecting the likelihood of outcome occurrence and receipt of study treatments with the aid of a causal diagram (Additional file 1: Fig. S1) [14]. For the present study, we identified two main potential sources of confounding: one being the degree of organ dysfunction in the first 8 h and two the “culture” of each participating ICU toward initiation of mechanical ventilation (see supplementary material). The logistic regression model used to construct the propensity score was:

Early intubation ~ age + PaO_2_/FiO_2_ + respiratory rate + PaCO_2_ + accessory muscle use + inability to clear secretions + non-respiratory SOFA + GCS + pH + vasopressor dose + Lactate + Platelet count + Creatinine + renal replacement + source of infection + median days of MV days by center + Intubation rate by center + Mortality rate by center.

Missing data on important confounders were handled using multiple imputation with a Monte Carlo Markov Chain method (see Additional file 1: Table S1) [15].

Our main analysis comprised a 1:1, nearest-neighbor propensity score matching without replacement [16]. Balance assessment was checked with the use of standardized mean differences (SMDs) in the covariates of interest, with smaller SMDs showing larger overlap in the adjusted populations. We considered a SMD cutoff of 0.2 to represent good adjustment. Because SMDs do not guarantee a similar covariate distribution when variables are not normally distributed, we also checked balance with the use of density plots [17, 18].

All analyses were carried out in the matched population. For in-hospital mortality, Kaplan–Meier curves were derived, and treatment effect was estimated through a semiparametric Cox model [19]. We did not formally test for Cox proportionality assumption, and we present hazard ratios as an average of treatment effect over the study time [20]. Patients were censored at hospital discharge or day 60 after study enrollment, whichever came first. Mortality was also assessed with Fisher’s exact test as well as the number of patients in whom treatment limitation orders had been in place. For ICU and hospital length of stay, non-respiratory and non-neurologic SOFA score, norepinephrine dose, lactate levels and time to onset to any treatment limitation order, we present medians (and differences) between groups. Fluid balance and the use of renal replacement were compared with regression. In order to construct 95% confidence intervals in the aforementioned analyses, we estimated standard errors with bootstrapping [21]. Significance was set at p value 0.05, considering a two-tailed distribution. No adjustment was carried out for multiple comparisons (see supplementary material).

### Sensitivity analyses

Several analyses were performed to assess the robustness of our findings. First, to assess if employing a different statistical method to deal with confounding by indication would affect the conclusions of our study, we performed overlap weighting, which assigns weights to patients based on their propensity score to create a pseudo-population with perfect overlapping in the probability of receiving the treatment at study (see supplementary material) [17]. Second, since clinicians normally rely on several markers that indicate the need for intubation, we repeated the study in a subset of patients who were displaying “traditional” intubation criteria. These criteria were defined as the presence of a Glasgow Coma Scale (GCS) of less than 10 or the presence of at least two of the following criteria: a ratio of PaO_2_/FiO_2_ less than 150, oxygen saturation less than 90% during more than 5 min despite optimized oxygen administration, a respiratory rate more than 35 breaths per minute, the presence of accessory muscle use and inability to clear secretions, pH less than 7.35 or PaCO_2_ above 45 mmHg. Third, we modified the exposure of interest to consider “early intubation” as the one occurring in the first 24 h since vasopressor start. Fourth, we included a complete-case analysis where patients with missing data were excluded. Finally, we performed an analysis of the effect of early intubation as compared to late intubation (beyond 8 h) on mortality, ICU and hospital length of stay (see supplementary material).

The R software (R Foundation for Statistical Computing, Vienna, Austria; packages boot, dplyr, finalfit, ggplot, ipw, lubridate, Matching, mice, tableone, sandwich and survey) was used throughout this study.

## Results

### Main analysis

**Fig. 1:**
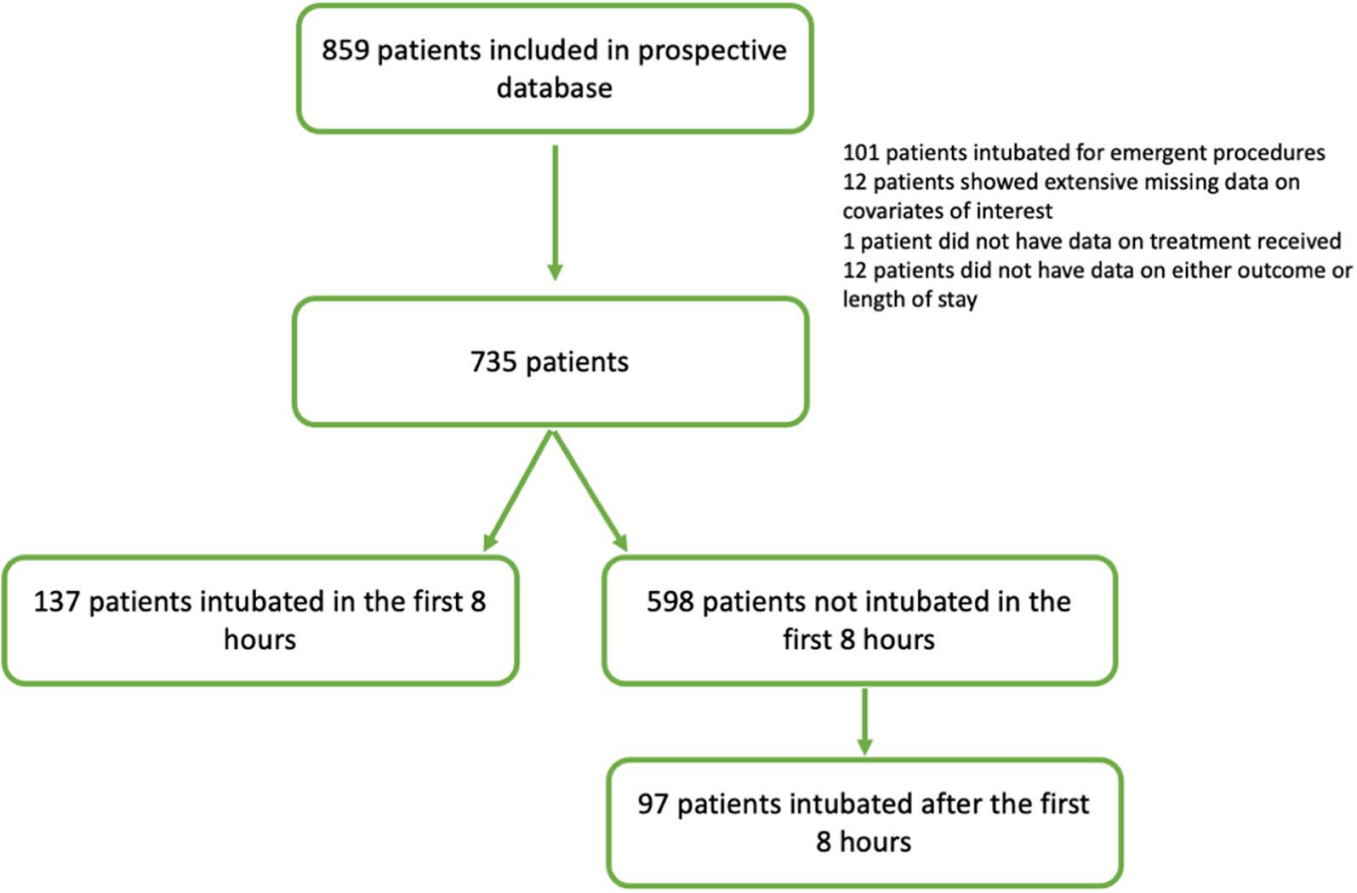
Study flowchart

Out of 859 patients included in the database, 112 were excluded since they had been intubated for interventional/surgical procedures, thus creating a final sample of 735 patients of whom 137 (19%) were intubated in the first 8 h at a median 2 h after vasopressor start (IQR 1–5 h). Out of the remaining 598 patients, 97 (16%) patients received intubation beyond 8 h (median 2nd day, 25–75% 1st–3rd day) (Fig. 1). The characteristics of the unadjusted population can be seen in Table 1, while the outcomes of this population can be checked in Additional file 1: Table S2.

**Table 1:** Distribution of covariates of interest in the unadjusted and weighted populations

Early intubation	Yes	No	*P* value	SMD	Yes	No	*P* value	SMD
Number of subjects	137	598			78	78		
Age	66 (15)	65 (14)	0.442	0.074	67 (14)	67 (13)	0.844	0.032
Body mass index	28 (6)	27 (7)	0.070	0.164	28 (6)	27 (7)	0.536	0.099
Neurological criteria for intubation, *n* (%)	37 (27)	6 (1)	< 0.001	0.26	3 (4)	4 (5)	1.000	0.062
Number of respiratory criteria for intubation			< 0.001				0.467	0.199
0–1	37 (27)	481 (80)		0.534	28 (36)	34 (44)		
2–3	66 (48)	107 (18)		0.303	39 (50.0)	37 (47)		
4–6	34 (25)	10 (2)		0.231	11 (14)	7 (9.0)		
Pulmonary sepsis, *n* (%)	48 (35)	137.0 (23)	0.003	0.121	30 (39)	24 (31)	0.400	0.162
Respiratory rate (rpm)	31 (8)	26 (7)	< 0.001	0.618	30 (7)	30 (7)	0.837	0.033
PaO_2_/FiO_2_	138 [85, 232]	275 [192, 381]	< 0.001	0.884	141 [89, 230]	153 [102, 232]	0.423	0.020
PaCO_2_ (mmHg)	37 (15)	32 (8)	0.001	0.388	34 (10)	33 (11)	0.765	0.048
Accessory muscle use, *n* (%)	89.0 (65.0)	70.0 (11.7)	< 0.001	0.533	43 (0.55)	36 (0.46)	0.337	0.179
Inability to clear secretions, *n* (%)	41.0 (29.9)	52.0 (8.7)	< 0.001	0.212	18 (0.23)	17 (0.22)	1.000	0.031
pH	7.25 (0.15)	7.37 (0.08)	< 0.001	1.005	7.31 (0.10)	7.31 (0.09)	0.967	0.007
Lactate (mmol/L)	4.3 [2.6, 6.6]	2.2 [1.6, 3.3]	< 0.001	0.811	3.5 [2, 4.5]	3 [2, 5.5]	0.487	0.071
Non-respiratory SOFA	9 [7–12]	8 [6–9]	< 0.001	0.706	8 [7, 10]	8 [7, 11]	0.970	0.032
Norepinephrine dose (mcg/kg/min)	0.59 [0.33, 1.00]	0.26 [0.15, 0.47]	< 0.001	0.790	0.50 [0.28, 0.77]	0.51 [0.26, 0.88]	0.762	0.120
Glasgow Coma Scale	14 [9, 15]	15 [15]	< 0.001	0.949	15 [14, 15]	15 [14, 15]	0.785	0.020
Platelet count, (10^12^/L)	170 (130)	170 (110)	0.953	0.006	160 (119)	151 (105)	0.614	0.081
Bilirubin, (mmol/L)	16 [9, 36]	15 [10, 27]	0.326	0.197	16 [9, 27]	15 [10, 25]	0.869	0.171
Creatinine, (mmol/L)	220 (172)	172 (153)	0.003	0.294	206 (161)	212 (184)	0.813	0.038
Renal replacement at T8	11 (8)	26 (4)	0.076	0.04	4 (5)	3 (4)	1.000	0.062
Median days of MV by center	6 [3, 7]	6 [3, 7]	0.666	0.004	5 [3, 7]	6 [3, 6]	0.727	0.139
Intubation rate by center (%)	0.22 [0.17, 0.33]	0.22 [0.17, 0.33]	0.514	0.038	0.21 [0.17, 0.32]	0.23 [0.17, 0.33]	0.600	0.072
Mortality rate by center (%)	0.23 [0.20, 0.29]	0.23 [0.20, 0.29]	0.726	0.039	0.23 [0.20, 0.29]	0.23 [0.20, 0.29]	0.801	0.007

**Fig. 2:**
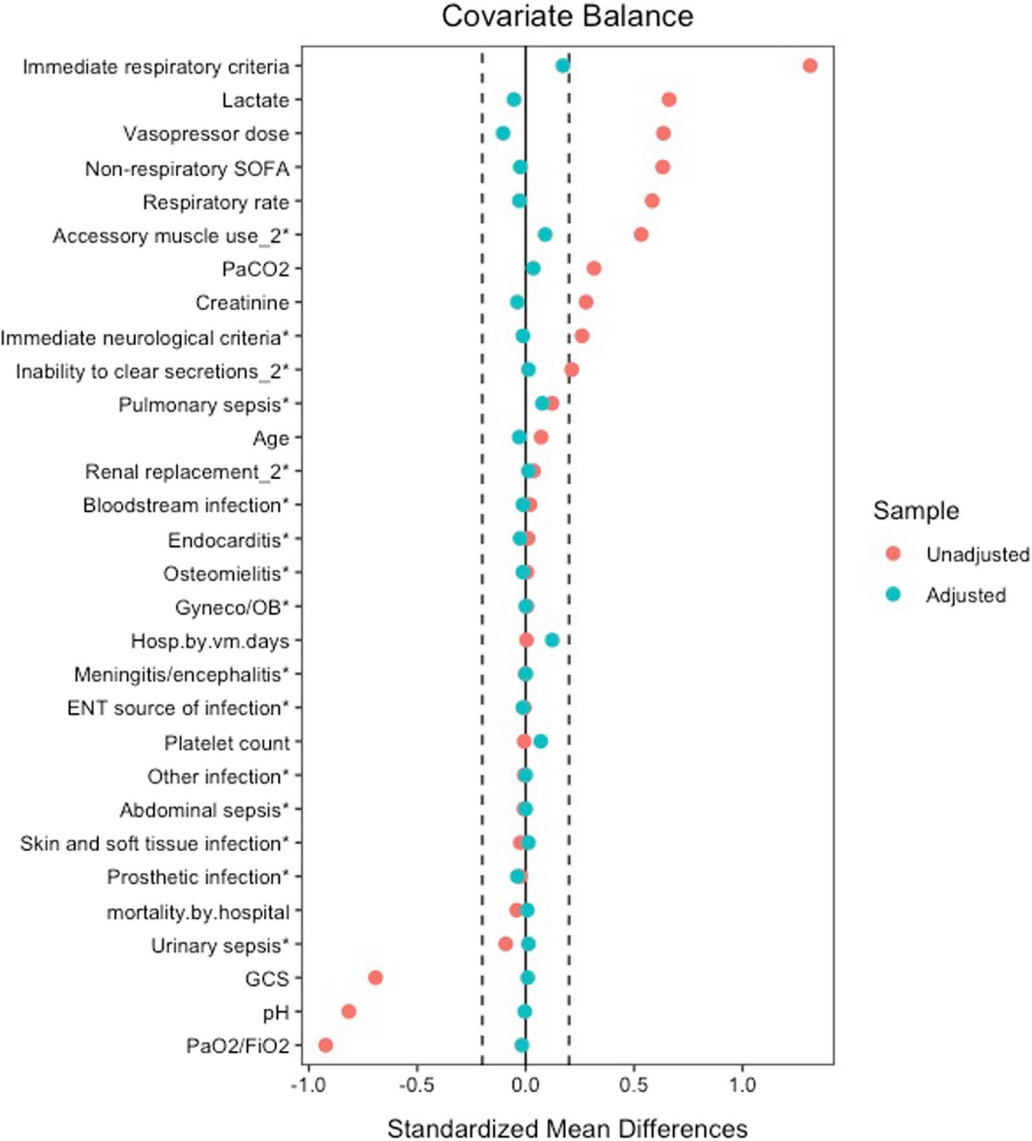
Covariate balance before and after matching. Dashed lines depict cutoff for good adjustment. Distance represents how far patients laid regarding the overall propensity score. *Categorical variables, where a difference in proportions was calculated instead of a standardized mean difference unlike Table 1 where all variable distances have been standardized

Propensity score matching led to a population comprised by 78 pairs, which represented 57% of those intubated in the first 8 h, with excellent covariate balance (Table 1; Fig. 2; Additional file 1: Fig. S2). Matched subjects among those intubated in the first 8 h showed less severity of disease when compared to unmatched individuals (Additional file 1: Table S3). Eventually, 27 (35%) subjects, who did not receive intubation in the first 8 h, underwent invasive mechanical ventilation during their ICU stay.

#### Primary outcome

**Fig. 3:**
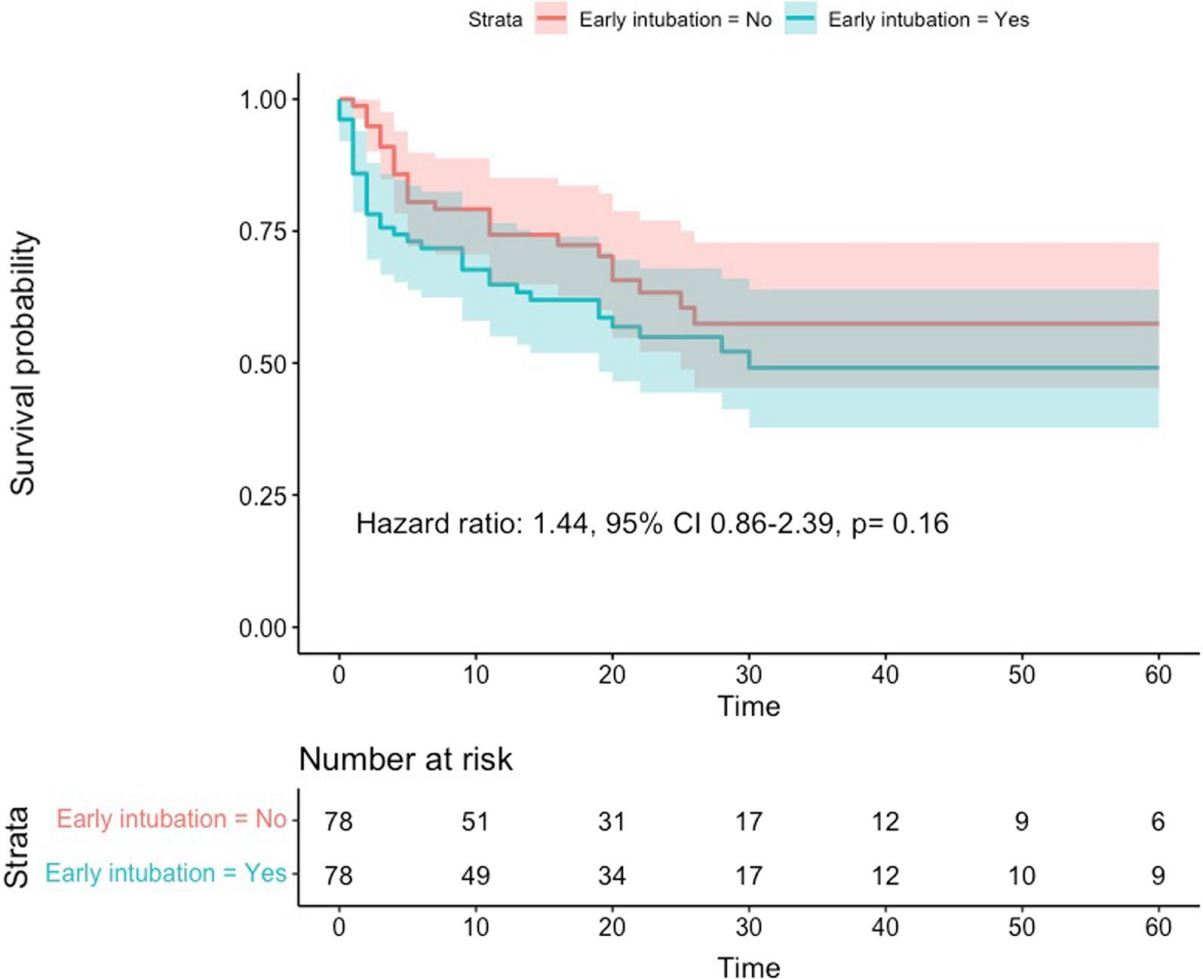
Kaplan–Meier curves and risk table to assess in-hospital mortality until day 60 after ICU admission for both study groups when treatment effect was assessed with propensity score matching. Confidence intervals are depicted in shaded areas. Time was assessed in days

In-hospital mortality occurred in 35 (45%) and in 26 (33%) subjects in the early intubation and unexposed groups (*p* = 0.19) [hazard ratio 1.44 95% CI 0.86–2.39, *p* = 0.16] (Fig. 3). Treatment limitation orders did not differ between groups 21 (27%) versus 16 (21%) [*p* = 0. 35], and these orders were often initiated at a similar time after vasopressor onset (median 5 vs. 4, 95% CI − 7 to 11 days).

#### Secondary outcomes

Early intubated subjects displayed a median ICU length of stay of 9 days, while subjects unexposed to intubation in the first 8 h spent a median of 5 days: (difference 4 days, 95% CI 1 to 7 days). Hospital stay was not different between groups: 14 versus 16 days (difference − 2 days, 95% CI − 7 to 8 days).

**Fig. 4:**
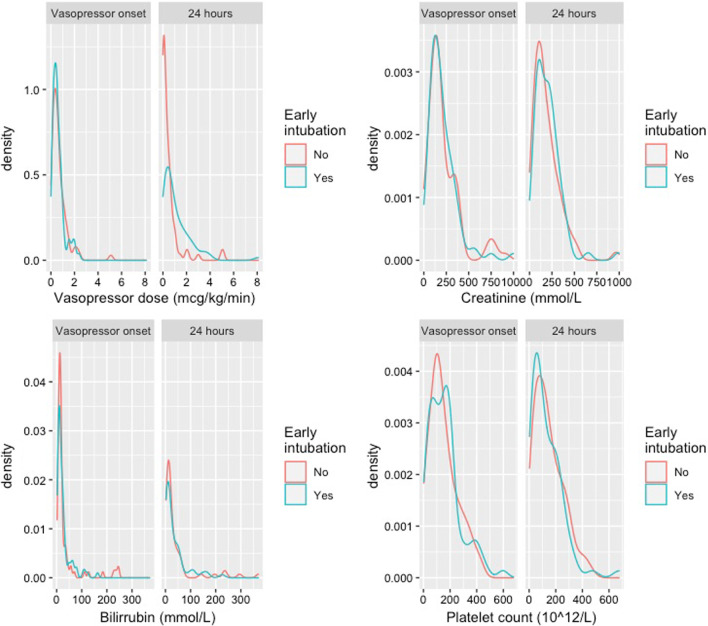
Non-respiratory, non-neurologic SOFA score components at vasopressor onset and at 24 h

We did not observe differences in non-respiratory, non-neurologic SOFA score at 24 h between groups (8 vs. 7 points, 95% CI − 1 to 3) (Fig. 4). Conversely, early intubated subjects received a higher median norepinephrine dose (0.69 vs. 0.22 mcg/kg/min, 95% 0.23–0.94), while lactate levels were not dissimilar (2.5 vs. 2.2 mmol/L, 95% CI − 0.6 to 2.7). Early intubated subjects presented with a more positive fluid balance (mean difference 2900 vs. 1050 mL, difference 1850 mL, 95% CI 1000–2600, *p* < 0.001). Additionally, renal replacement techniques at 24 h were also used more frequently in this group (38% vs. 15%, 95% CI 8–36, *p* = 0.001).

### Sensitivity analysis

Further analyses were carried out with overlap weighting, by restricting the population to those showing intubation criteria only, by modifying the exposure time or conducted on a complete-case analysis (Additional file 1: Tables S4–S7 and Figs. S3–S5).

**Fig. 5:**
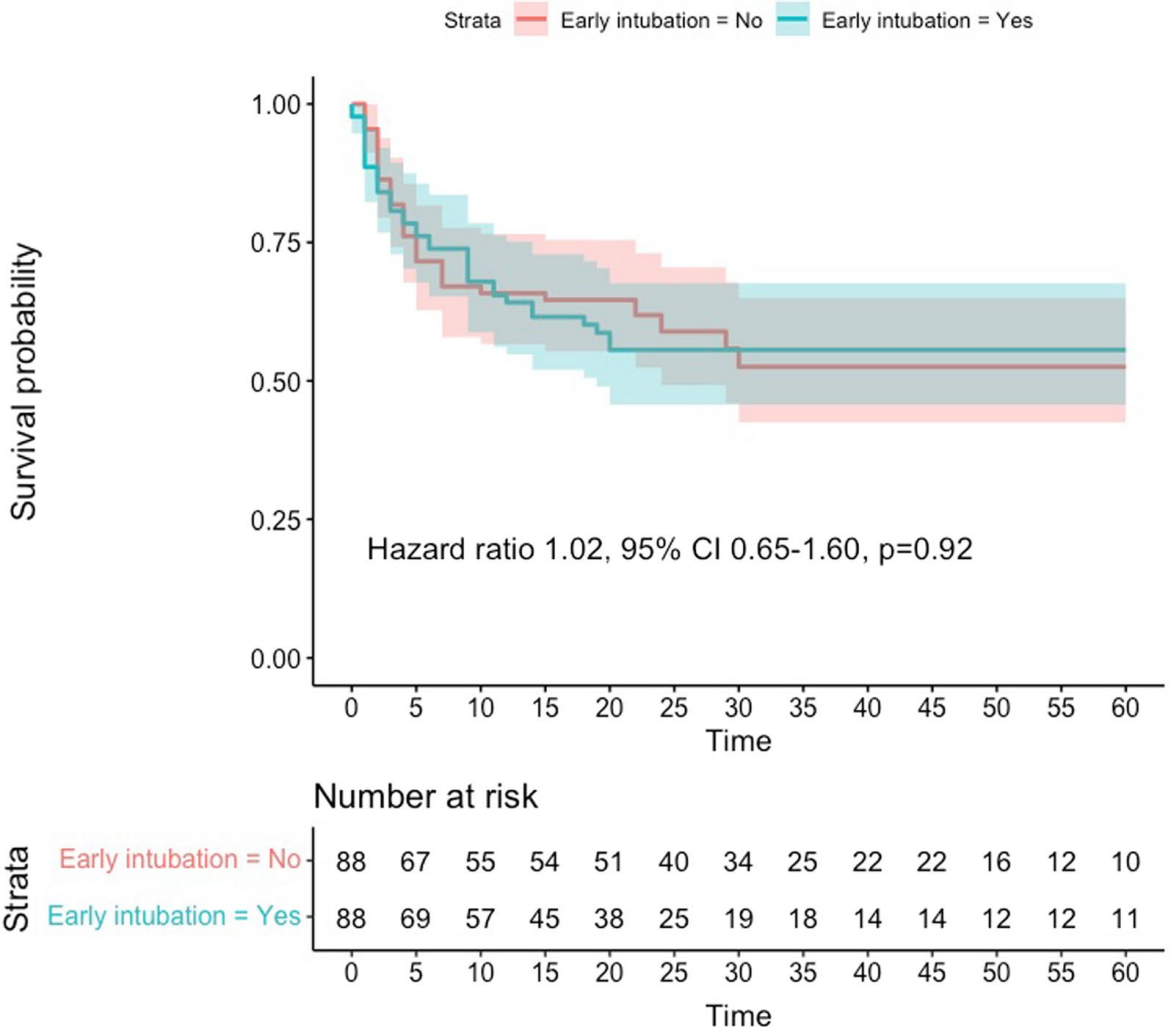
Comparison between patients who were intubated early or late (beyond 8 h) using propensity score matching. Confidence intervals are depicted in shaded areas. Time was assessed in days

Finally, by including only patients who received mechanical ventilation, we identified 234 subjects: 137 in the first 8 h and 97 beyond the 9th hour. Matching identified 88 pairs with good adjustment (Additional file 1: Table S8). In this analysis, mortality occurred in 37 and 39 individuals, respectively (hazard ratio 1.02, 95% CI 0.65–1.90, *p* = 0.92) (Fig. 5). An additional analysis with overlap weighting did not show different results (Additional file 1: Table S9; Fig. S6). A summary of the results from these secondary analyses can be checked in Additional file 1: Table S10.

## Discussion

This study examined the effect of early intubation in septic shock patients on in-hospital mortality, intensive care and hospital length of stay and non-respiratory and neurologic organ dysfunction at 24 h compared to a strategy of no intubation in the same time period. Specifically, we assessed the effects of intubation in the first 8 h after vasopressor start after adjusting for potential confounders. The main finding of this study was that early intubation was not associated to any benefit in terms of in-hospital mortality. Several sensitivity analyses showed an increased signal for harm; nonetheless, these findings were non-consistent throughout the methods used (Fisher’s test vs. Kaplan–Meier). We believe that, given the observational nature of this study and the multiple tests we performed, these observations should be interpreted with extreme caution and probably considered to represent a true “null effect” rather a negative effect for intubation. ICU length of stay was significantly longer in subjects intubated in the first 8 h, while hospital stay was not. Early intubated subjects showed, on average, a more positive fluid balance at 24 h and were started more often on renal replacement.

Previous research has shown that patients who are intubated late show a worse prognosis than those intubated early in their course. This observation was noted even when intubation was delayed for as little as 6 h [10, 22]. Although this finding has been described mostly in acute respiratory failure, septic shock is not an exclusive diagnosis and often patients in our population presented with notable hypoxemia. Indeed, in one-third of them, the source of infection was pulmonary in origin. Although these studies point toward a causal negative effect caused by a delay in intubation, presumably through the harm caused by vigorous spontaneous breathing, which has been experimentally shown to increase lung injury in primed organs [11, 23], it is noteworthy to remind that these studies did not take into account patients in whom intubation was spared. In other words, while delaying intubation could be deleterious per se, avoiding intubation in a significant number of patients may possibly offset the benefits of the former, at least in a subset of patients with a less severe disease. In our study, when patients intubated late were compared to those intubated early, mortality was unchanged between groups. If this differential finding as compared to previous research is related to a lesser degree of lung injury cannot be confirmed at this point [24]. In the present study, 35% of subjects who were not intubated in the first 8 h ended up receiving mechanical ventilation, which is slightly less frequent than in previous studies in patients with acute respiratory failure [4, 5]. Our findings are in line with a matched analysis in COVID-19 individuals and a meta-analysis of observational studies in COVID-19 [25, 26].

The INTUBATIC study showed that only half of the patients displaying theoretical criteria for immediate intubation received mechanical ventilation [12]. An interesting finding of the present work is that when restricting the analysis to patients who were displaying these criteria only, a subpopulation of sicker patients that could theoretically benefit from early intubation, the results did not differ from the main analysis. Although these criteria were arbitrarily defined, clinicians will likely recognize factors commonly used in daily practice to decide if intubation should be pursued. Moreover, the same or similar criteria have been previously used in clinical trials to assess the efficacy of noninvasive respiratory support devices [4, 5].

Several considerations must be considered in the interpretation of the results of this study. Most importantly, since baseline characteristics largely differed between groups, to estimate treatment effects we adjusted for an extensive list of confounders which were selected using a causal diagram. Also, given this large imbalance, assessing the outcome in the whole population was unfeasible and we targeted our analysis at a population where clinicians most likely would have found difficulties in deciding whether intubation had to be carried out [17]. Specifically, we were able to match from 32 to 64% (57% for the principal analysis) of the patients who received early intubation and up to 91% (88 out of 97) of those who underwent intubation in a later period. That is to say that we did not assess those patients who were too sick at inclusion and in whom intubation had likely been life-saving or those individuals intubated late who presented with milder disease at admission but deteriorated thereafter. These observations are essential, in our opinion, to understand that the findings of this research do not point at invalidating the usefulness of mechanical ventilation in septic shock patients but rather at questioning the use of an early approach in patients with baseline characteristics as those displayed in the adjusted populations. Third, we considered an 8-h window to define what early intubation meant. Although this is arbitrary, the INTUBATIC study had previously used this time frame [12]. Moreover, previous research has shown that delaying intubation for just a few hours could be deleterious [10]. In a sensitivity analysis, we expanded this definition to the first 24 h without showing discrepant results. Fourth, it is important to consider that covariate adjustment was carried out based on the most abnormal values in the first 8 h. However, intensivists not only consider the degree of physiological abnormality but also its change over time to decide if intubation is needed. Since the INTUBATIC database included only one value in each timepoint of interest, considering the dynamic component of acute disease was not possible. Although this could represent a source of bias, it must be emphasized that treated subjects were intubated at a median of 2 h after vasopressor start, a factor that would likely have minimized the imprecision created by the impossibility to model the time-varying nature of the decision-making process. Finally, we were unable to provide data on patient complications such as ventilator-associated pneumonia or delirium.

To sum up, the results of this work might suggest that in septic shock patients with similar characteristics to those shown in the adjusted populations of this study, a wait-and-see strategy toward intubation with noninvasive support as a first treatment step could decrease the use of intubation without incurring in an excess of mortality. Although not assessed in this study, when confronted with higher disease severity, expeditious intubation might be necessary.

## Conclusions

Intubation in the first 8 h after vasopressor start did not confer a benefit in terms of mortality in this matched cohort of septic shock patients. After this timepoint, late intubation was needed in one-third of patients. Late intubation was not associated with increased mortality. This analysis could only assess half of those individuals who were intubated in the first 8 h because of large baseline imbalances between early intubated subjects and non-intubated patients. These findings may have important clinical implications but should be confirmed in future trials.

## Supplementary Information

**Additional file 1.** Additional information on Methods. Additional Results.

## Data Availability

The datasets used and analyzed during the current study are available from the corresponding author of reasonable request.

## Code availability

The statistical analysis performed is available upon request.
