# Supplementary material for: Early intubation and patient-centered outcomes in septic shock: a secondary analysis of a prospective multicenter study
Source: Crit Care. 2022 Jun 7;26:163. doi: 10.1186/s13054-022-04029-6 (PMC9171484; doi:10.1186/s13054-022-04029-6)
Supplement: Supplementary file 1 — Additional file 1. Additional information on Methods. Additional Results. [file 13054_2022_4029_MOESM1_ESM.docx]

**Early Intubation and patient-centered outcomes in septic shock: a secondary analysis of a prospective multicenter study**

*Running title: Early intubation in septic shock*

Ricard Mellado-Artigas (MD)^1,2^

Carlos Ferrando (MD, PhD)^1,2^

Frédéric Martino (MD)^3^

Agathe Delbove (MD)^4^

Bruno L. Ferreyro (MD)^5,6^

Cedric Darreau (MD)^7^

Sophie Jacquier (MD)^8^

Laurent Brochard (MD, PhD)^6,9^

Nicolas Lerolle (MD, PhD)^10^

From

1. *Surgical Intensive Care Unit, Hospital Clínic, Institut D'investigació August Pi i Sunyer, Barcelona, Spain*
2. *CIBER de Enfermedades Respiratorias, Instituto de Salud Carlos III, Madrid, Spain*
3. *Medical and Surgical Intensive Care Unit, Guadeloupe University Hospital, Les Abymes, Guadeloupe, France*
4. *Medical Intensive Care Unit, Nantes University Hospital, Nantes, France*
5. *Department of Medicine, Sinai Health System and University Health Network, Toronto, Canada*
6. *Interdepartmental Division of Critical Care Medicine, University of Toronto, Canada*
7. *Medical and Surgical Intensive Care Unit, Le Mans Hospital, Le Mans, France*
8. *Medical Intensive Care Unit, Tours University Hospital, Tours, Franc*
9. *Keenan Research Centre for Biomedical Science at the Li Kan Shing Knowledge Institute, St Michael’s Hospital, Toronto, Ontario, Canada*
10. *Medical Intensive Care Unit, Angers University Hospital and Angers Faculty for Health Sciences, Angers, France*

**Corresponding Author:**

Ricard Mellado-Artigas. Surgical ICU (Department of Anaesthesiology), Hospital Clinic. Villarroel 170, 08025, Barcelona, Spain.

Phone: +34 932275558. Email: rmartigas@gmail.com

ORCID number: 0000-0003-2815-6819

**INDEX**

[ADDITIONAL INFORMATION ON METHODS: 3](#_Toc103621939)

[*Multiple imputation:* 4](#_Toc103621940)

[*Standard errors and confidence intervals:* 6](#_Toc103621941)

[ADDITIONAL RESULTS: 7](#_Toc103621942)

[TABLES 7](#_Toc103621943)

[Table S2: Outcomes in the unadjusted population.. 8](#_Toc103621944)

[Table S3: Characteristics of matched and unmatched exposed subjects (intubated in the first 8 hours). 8](#_Toc103621945)

[Table S4: Distribution of covariates of interest in the weighted population.. 9](#_Toc103621946)

[Table S5: Weighted population targeting patients with traditional criteria for intubation.. 10](#_Toc103621947)

[Table S6: Weighted population after modifying exposure time to 24 hours. 11](#_Toc103621948)

[Table S7: Weighted population in the complete-case population. 12](#_Toc103621949)

[Table S8: Matched population when comparing early and late intubation using an 8-hour cutoff 13](#_Toc103621950)

[Table S9: Weighted population (using overlap weighting) when comparing early and late intubation using an 8-hour cutoff 14](#_Toc103621951)

[Table S10: Outcomes in the sensitivity analyses. Early intubated subjects vs unexposed individuals.](#_Toc103621952)

[the differences are depicted within parentheses. 15](#_Toc103621952)

[FIGURES 17](#_Toc103621953)

[*Figure S1: Direct acyclic graph (DAG) for the assessment of early intubation on mortality, ventilator-free days (VFD) and ICU length of stay.* 18](#_Toc103621954)

[*Figure S2: Distribution of covariates in the matched population.* 19](#_Toc103621955)

[*Figure S3: Kaplan Meier curves when the adjusted population was created with overlap weighting.* 20](#_Toc103621956)

[*Figure S4: Kaplan Meier curves for the comparison of patients displaying immediate criteria for intubation.* 21](#_Toc103621957)

[*Figure S4: Kaplan Meier curves for the comparison of patients after modifying exposure up to 24 hours.* 22](#_Toc103621958)

[*Figure S5: Kaplan Meier curves for the comparison of patients after carrying out a complete-case analysis.* 23](#_Toc103621959)

[*Figure S6: Comparison between patients who were intubated early or late (beyond 8 hours) using overlap weighting.* 24](#_Toc103621960)

[REFERENCES: 25](#_Toc103621961)

# **ADDITIONAL INFORMATION ON METHODS:**

*Covariate selection:*

Covariate adjustment poses the risk to induce selection bias if conditioning is performed on variables that act as colliders. The structural relationship between the exposure of interest and outcome, including all other potential covariates can be depicted using a direct acyclic graph (DAG). This form of causal diagram is constructed from expert knowledge including previous research and describes the roadmap of causal connections between variables of interest. Through the assessment of relationships between variables, DAGs help to identify potential sources of bias and how to control for them when estimating causal associations^1^. For the present study, we created one DAG that identified two key elements to be controlled for: 1) organ dysfunction and 2) ICU culture. Organ dysfunction was measured by surrogates of respiratory and non-respiratory involvement. Likewise, we estimated the effect of ICUs (ICU culture) by creating three variables; namely the rate of intubation by each center, the median days an intubated individual would spend ventilated by center and, finally, the mortality rate by center.

Based on the DAG drawn, the adjustment needed would include only surrogate variables for organ dysfunction and ICU culture. However, since DAGs represent an attempt to reflect reality, but researchers are never fully certain of the model depicted, we considered that further controlling for age, body mass index and the source of infection could decrease bias.

## *Multiple imputation:*

Missing data occurs commonly in observational research. This limits the ability to adjust for confounding since it reduces sample size when an ambitious adjustment is pursued. To avoid this problem, we carried out multiple imputation with Monte Carlo Markov chain methods using the *mice* package in R^2,3^. After removing 101 patients who were intubated for invasive procedures or surgery and 25 patients that had either extensive missing data (>50% of the variables of interest) or missing data on the treatment or outcome, 735 remained in the database. Since the main goal of this study was to estimate the effect of intubation in the first 8 hours after ICU admission, minimizing missing data on predictors’ values that occurred before intubation was essential. Based on the causal diagrams used for this research, we identified variables linked to organ dysfunction as to represent key elements, thus, we aimed at “filling blanks” only on the predictors of interest based on the other predictors of interest or demographic data such as age, gender or body mass index. We did not include treatment assignment nor outcomes to predict missing values, nor we imputed values on these variables.

Specifically, for the variables of interest connected to organ dysfunction in the first 8 hours of study; 309 subjects (42%) showed at least 1 missing value. Median missing values per subject was 0 (IQR 0-1) and 20 subjects presented with missing data on >50% of the predictors and were excluded, thus, leaving a final sample comprised of 735 patients. Median percentage of missing values per variable was 4% (IQR 1-14%) while the total number of missing values were 733, which represented 5.5% of the total number of cells (Table S1). Non-respiratory SOFA was not imputed when missing but rather recalculated with the imputed items included in its definition.

Additionally, since the INTUBATIC registry offered information on data collected from the ninth hour to the end of day 1, and in order to assess the change in SOFA score between the first eight hours and 24 hours, a second multiple imputation was carried out. This two-step process was done to avoid values collected at 24 hours to influence imputed values on variables collected in the first 8 hours. Finally, fluid balance and the use of continuous renal replacement were not imputed. Fluid balance did not include the extraction achieved by renal replacement when this technique was used.

*Logistic regression (propensity score):*

We pursued covariate adjustment based on the propensity score, which is defined as the probability of receiving the treatment of interest. In this model, it is also recommended to include variables that, despite not being causally associated with the exposure, are linked to the outcome^4^. As mentioned before, covariate selection was done based on a DAG. The logistic regression model used in this study for all the analyses was:

Early intubation ~age + PaO_2_/FiO_2_ + respiratory rate + PaCO_2_ + accessory muscle use + inability to clear secretions + non-respiratory SOFA + GCS + pH + vasopressor dose + Lactate + Platelet count + Creatinine + renal replacement + source of infection + MV days by centre + Intubation rate by centre + Mortality by centre.

*Propensity score matching:*

Once the logistic regression model had been constructed, the probability of receiving the treatment (the propensity score) was calculated. This value was later used to create matches, using propensity score matching (PSM). For this matter, we used a 1:1 nearest-neighbour matching strategy without replacement with the calliper set at 0.1 or 0.2 standard deviations^4^.

*Overlap weighting:*

The propensity score was used to create weights, using overlap weighting, a statistical tool that constructs a pseudo-population with exact covariate distribution. With this method, individuals are weighted by the probability of receiving the opposite treatment, which leads to excellent adjustment but at the cost of reducing the pseudo-population sample size. Although, this method shows some similarities with matching, overlapping subjects are never thrown out in case the number of treated subjects exceeds the number of controls (a fact that can be overcome in matching with replacement at the cost of increasing variance). For all these reasons, this weighting method can be used to make inferences in the overlapping population, where clinicians are most interested in assessing treatment effect^5^.

## *Standard errors and confidence intervals:*

Naïve standard errors as provided by most statistical software are valid only when certain assumptions are met. Nonetheless, in many situations these conditions are violated, thus leading to standard errors to underestimate the true variance^6^. For this reason, in this study, standard errors of coefficients of interest were estimated with non-parametric bootstrapping after creating at least 200 replications. Standard errors were constructed as the standard deviation of the bootstrapped estimates. A histogram was plot and normality checked with Shapiro-Wilk test. If normality was confirmed, 95% confidence intervals were calculated as the original coefficient mean minus the bias plus/minus 1.96* bootstrapped standard error. In case, the distribution was not considered to be normal, we estimated the mean coefficient as the median of the bootstrapped estimated. 95% confidence intervals were calculated as 2.5 and 97.5 percentiles, accordingly.

*Sensitivity analysis:*

To assess the robustness of our findings, we performed changes in our analysis to estimate the effect with either a different statistical tool (overlap weighting) or in modified populations. We also aimed at comparing patients who were intubated in the first 8 hours to those intubated afterwards. Except for the analysis with overlap weighting in the whole population, our target was to estimate treatment effect with propensity score matching. However, the degree of adjustment was poor for several important variables, such as PaO_2_/FiO_2_ or pulmonary sepsis as source of infection and we decided to present these sensitivity analyses using overlap weighting when balance achieved with matching was inadequate. For the analysis comparing early to late intubation, we present both assessments.

# **ADDITIONAL RESULTS:**

## **TABLES**

Table S1: Missing data per variable included in the propensity score estimation. No missing data was present for center-derived variables.

| **Variable** | **Number and percentage of missing values** |
| --- | --- |
| Age | 3/735 (<1%) |
| PaO_2_/FiO_2_ | 151/735 (21%) |
| Respiratory rate | 30/735 (4%) |
| PaCO_2_ | 109/735 (15%) |
| Accessory muscle use | 12/735 (2%) |
| Inability to clear secretions | 11/735 (2%) |
| Non-respiratory SOFA | 129/735 (18%) |
| GCS | 5/735 (<1%) |
| pH | 105/735 (14%) |
| Vasopressor dose | 6/735 (<1%) |
| Lactate | 111/735 (15%) |
| Platelet count | 39/735 (5%) |
| Creatinine | 21/735 (3%) |
| Renal replacement | 1/735 (<1%) |
| Source of infection | 0/735 (<1%) |

### Table S2: Outcomes in the unadjusted population. P-value for mortality assessed by unadjusted Cox model and Wilcoxon’s sum ranked test for ICU and hospital length of stay.

| **Early intubation**  **Number of subjects** | **Yes**  **137** | **No**  **598** | **P-value** |
| --- | --- | --- | --- |
| Intubation beyond 8 hours | - | 97 (16%) | - |
| Mortality by day 60 | 76 (55%) | 111 (19%) | <0.001 |
| ICU length of stay | 7 | 4 | 0.003 |
| Hospital length of stay | 12 | 13 | 0.001 |

### Table S3: Characteristics of matched and unmatched exposed subjects (intubated in the first 8 hours).

| **Early intubation** | **Yes** | **No** | **p-value** | **SMD** |
| --- | --- | --- | --- | --- |
| **Number of subjects** | **78** | **59** |  |  |
| Age | 66.85 (14.22) | 64.63 (15.27) | 0.383 | 0.150 |
| Body mass index | 27.75 (6.10) | 27.78 (5.88) | 0.978 | 0.005 |
| Neurological criteria, n (%) | 0.04 (0.19) | 0.58 (0.50) | <0.001 | 1.434 |
| Number of respiratory criteria* |  |  | <0.001 | 0.670 |
| 0-1 | 20 (25.6) | 5 ( 8.5) |  |  |
| 2-3 | 45 (57.7) | 26 (44.1) |  |  |
| 4-6 | 13 (16.7) | 28 (47.5) |  |  |
| Pulmonary sepsis, n (%) | 30 (38.5) | 18 (30.5) | 0.432 | 0.168 |
| Respiratory rate (rpm) | 29.83 (6.56) | 31.75 (8.73) | 0.146 | 0.248 |
| PaO_2_/FiO_2_ | 174.41 (111.86) | 176.87 (129.67) | 0.906 | 0.020 |
| PaCO_2_ (mmHg) | 33.87 (10.42) | 40.73 (18.24) | 0.006 | 0.462 |
| Accessory muscle use, n (%) | 43 (55.1) | 46 (78.0) | 0.009 | 0.499 |
| Inability to clear secretions, n (%) | 18 (23.1) | 23 (39.0) | 0.068 | 0.349 |
| pH | 7.31 (0.10) | 7.18 (0.16) | <0.001 | 0.989 |
| Lactate (mmol/L) | 3.99 (2.20) | 7.01 (4.62) | <0.001 | 0.833 |
| Non-respiratory SOFA | 8.74 (2.33) | 11.10 (3.43) | <0.001 | 0.804 |
| Norepinephrine dose (mcg/kg/min) | 0.61 (0.49) | 1.11 (0.85) | <0.001 | 0.716 |
| Glasgow Coma Scale | 13.96 (1.80) | 8.98 (4.54) | <0.001 | 1.443 |
| Platelet count, (10^12/L) | 159.56 (118.81) | 182.66 (143.75) | 0.305 | 0.175 |
| Bilirubin, (mmol/L) | 26.24 (29.65) | 60.20 (107.12) | 0.009 | 0.432 |
| Creatinine, (mmol/L) | 205.96 (160.90) | 238.99 (184.89) | 0.267 | 0.191 |
| Renal replacement at T8 | 4 ( 5.1) | 7 (11.9) | 0.263 | 0.243 |
| Median days of MV by center | 5.60 (3.54) | 5.95 (3.62) | 0.568 | 0.099 |
| Intubation rate by center (%) | 0.25 (0.13) | 0.25 (0.12) | 0.769 | 0.051 |
| Mortality rate by center (%) | 0.24 (0.08) | 0.25 (0.09) | 0.847 | 0.033 |

### Table S4: Distribution of covariates of interest in the weighted population. Continuous variables are expressed as mean and standard deviation or median and interquartile range.

| **Early intubation** | **Yes** | **No** | **SMD** |
| --- | --- | --- | --- |
| **Number of subjects** | **54** | **54** |  |
| Age | 66.97 (14.82) | 66.97 (14.82) | <0.001 |
| Body mass index | 27.21 (5.72) | 27.21 (5.72) | 0.001 |
| Neurological criteria, n (%) | 0.06 (0.24) | 3.2 ( 5.9) | 0.006 |
| Number of respiratory criteria |  |  | 0.036 |
| 0-1 | 23.5 (43.8) | 17.1 (31.9) |  |
| 2-3 | 24.1 (45.0) | 29.2 (54.4) |  |
| 4-6 | 6.0 (11.2) | 7.3 (13.7) |  |
| Pulmonary sepsis, n (%) | 18.3 (34.1) | 18.3 (34.1) | <0.001 |
| Respiratory rate (rpm) | 29.57 (6.94) | 29.57 (6.94) | <0.001 |
| PaO_2_/FiO_2_ | 190.92 (122.57) | 160.00 [91.68, 267.68] | <0.001 |
| PaCO_2_ (mmHg) | 34.02 (11.20) | 34.02 (11.20) | <0.001 |
| Accessory muscle use, n (%) | 24.9 (46.5) | 24.9 (46.5) | <0.001 |
| Inability to clear secretions, n (%) | 10.2 (19.1) | 10.2 (19.1) | <0.001 |
| pH | 7.32 (0.10) | 7.32 (0.10) | <0.001 |
| Lactate (mmol/L) | 4.12 (2.35) | 3.50 [2.30, 5.10] | <0.001 |
| Non-respiratory SOFA | 8.78 (2.38) | 8.00 [7.00, 10.00] | <0.001 |
| Norepinephrine dose (mcg/kg/min) | 0.62 (0.52) | 0.50 [0.28, 0.79] | <0.001 |
| Glasgow Coma Scale | 13.90 (2.07) | 15.00 [14.00, 15.00] | <0.001 |
| Platelet count, (10^12/L) | 158.89 (118.05) | 158.89 (118.05) | <0.001 |
| Bilirubin, (mmol/L) | 27.78 (32.94) | 16.00 [10.00, 27.59] | 0.156 |
| Creatinine, (mmol/L) | 199.94 (138.51) | 199.94 (138.51) | <0.001 |
| Renal replacement at T8 | 1.03 (0.17) | 1.5 ( 2.8) | <0.001 |
| Median days of MV by center | 5.57 (3.53) | 4.13 [3.00, 7.00] | <0.001 |
| Intubation rate by center (%) | 0.25 (0.12) | 0.21 [0.17, 0.32] | 0.005 |
| Mortality rate by center (%) | 0.24 (0.08) | 0.23 [0.20, 0.27] | <0.001 |

### Table S5: Weighted population targeting patients with traditional criteria for intubation. Continuous variables are expressed as mean and standard deviation or median and interquartile range.

| **Early intubation** | **Yes** | **No** | **SMD** |
| --- | --- | --- | --- |
| **Number of subjects** | **29** | **29** |  |
| Age | 66.08 (14.35) | 66.08 (14.61) | <0.001 |
| Body mass index | 27.80 (5.74) | 27.28 (6.39) | 0.087 |
| Neurological criteria, n (%) | 2.9 ( 9.9) | 3.0 (10.3) | 0.013 |
| Number of respiratory criteria* |  |  | 0.044 |
| 0-1 | 1.1 ( 3.8) | 1.2 ( 4.2) |  |
| 2-3 | 21.8 (74.9) | 22.1 (76.2) |  |
| 4-6 | 6.2 (21.3) | 5.7 (19.6) |  |
| Pulmonary sepsis, n (%) | 13.1 (45.1) | 13.1 (45.1) | <0.001 |
| Respiratory rate (rpm) | 30.57 (7.15) | 30.57 (6.85) | <0.001 |
| PaO_2_/FiO_2_ | 114.16 [80.00, 216.85] | 116.42 [92.17, 193.70] | <0.001 |
| PaCO_2_ (mmHg) | 36.45 (12.50) | 36.45 (11.39) | <0.001 |
| Accessory muscle use, n (%) | 19.4 (66.7) | 19.4 (66.7) | <0.001 |
| Inability to clear secretions, n (%) | 8.2 (28.3) | 8.2 (28.3) | <0.001 |
| pH | 7.29 (0.09) | 7.29 (0.08) | <0.001 |
| Lactate (mmol/L) | 3.20 [2.33, 5.10] | 3.10 [1.90, 5.21] | <0.001 |
| Non-respiratory SOFA | 8.00 [7.00, 10.00] | 8.00 [7.00, 10.00] | <0.001 |
| Norepinephrine dose (mcg/kg/min) | 0.50 [0.26, 0.81] | 0.50 [0.25, 0.74] | <0.001 |
| Glasgow Coma Scale | 15.00 [14.00, 15.00] | 15.00 [14.00, 15.00] | <0.001 |
| Platelet count, (10^12/L) | 162.07 (116.18) | 162.07 (102.41) | <0.001 |
| Bilirubin, (mmol/L) | 13.02 [9.00, 23.40] | 15.00 [10.00, 22.03] | 0.041 |
| Creatinine, (mmol/L) | 191.24 (136.25) | 191.24 (133.04) | <0.001 |
| Renal replacement at T8 | 1.2 ( 4.0) | 1.2 ( 4.0) | <0.001 |
| Median days of MV by center | 5.00 [3.00, 6.28] | 4.00 [3.00, 7.00] | <0.001 |
| Intubation rate by center (%) | 0.20 [0.17, 0.32] | 0.24 [0.17, 0.32] | 0.101 |
| Mortality rate by center (%) | 0.21 [0.20, 0.27] | 0.23 [0.20, 0.29] | <0.001 |

### Table S6: Weighted population after modifying exposure time to 24 hours. Continuous variables are expressed as mean and standard deviation or median and interquartile range.

| **Early intubation** | **Yes** | **No** | **SMD** |
| --- | --- | --- | --- |
| **Number of subjects** | **67** | **67** |  |
| Age | 66.59 (14.40) | 66.59 (15.18) | <0.001 |
| Body mass index | 27.66 (5.99) | 26.49 (6.23) | 0.192 |
| Neurological criteria, n (%) | 4.9 ( 7.3) | 4.7 ( 7.0) | 0.012 |
| Number of respiratory criteria |  |  | <0.001 |
| 0-1 | 25.9 ( 38.8) | 25.9 ( 38.8) |  |
| 2-3 | 34.0 ( 51.0) | 34.0 ( 51.0) |  |
| 4-6 | 6.8 ( 10.2) | 6.8 ( 10.2) |  |
| Pulmonary sepsis, n (%) | 22.9 ( 34.4) | 22.9 ( 34.4) | <0.001 |
| PaO_2_/FiO_2_ | 29.84 (6.62) | 29.84 (7.07) | <0.001 |
| Respiratory rate (rpm) | 160.56 [90.97, 269.57] | 163.89 [101.44, 259.38] | <0.001 |
| PaCO_2_ (mmHg) | 34.84 (12.60) | 34.84 (10.54) | <0.001 |
| Accessory muscle use, n (%) | 66.7 (100.0) | 66.7 (100.0) | <0.001 |
| Inability to clear secretions, n (%) | 66.7 (100.0) | 66.7 (100.0) | <0.001 |
| pH | 7.31 (0.10) | 7.31 (0.09) | <0.001 |
| Lactate (mmol/L) | 3.50 [2.30, 5.00] | 2.90 [1.90, 5.20] | <0.001 |
| Non-respiratory SOFA | 8.00 [7.00, 10.00] | 8.00 [7.00, 10.00] | <0.001 |
| Norepinephrine dose (mcg/kg/min) | 0.50 [0.29, 0.85] | 0.44 [0.22, 0.75] | <0.001 |
| Glasgow Coma Scale | 15.00 [14.00, 15.00] | 15.00 [14.00, 15.00] | <0.001 |
| Platelet count, (10^12/L) | 167.20 (123.94) | 167.20 (110.12) | <0.001 |
| Bilirubin, (mmol/L) | 16.00 [10.00, 27.31] | 17.00 [12.00, 33.00] | 0.178 |
| Creatinine, (mmol/L) | 201.04 (146.69) | 201.04 (168.65) | <0.001 |
| Renal replacement at T8 | 2.3 (3.4) | 2.3 (3.4) | <0.001 |
| Median days of MV by center | 6.00 [3.00, 7.00] | 6.00 [3.00, 7.00] | 0.013 |
| Intubation rate by center (%) | 0.22 [0.17, 0.32] | 0.22 [0.17, 0.33] | <0.001 |
| Mortality rate by center (%) | 0.23 [0.20, 0.27] | 0.23 [0.20, 0.27] | <0.001 |

### Table S7: Weighted population in the complete-case population. Continuous variables are expressed as mean and standard deviation or median and interquartile range. Weighted populations allow for fractions of subjects to be created.

| **Early intubation** | **Yes** | **No** | **SMD** |
| --- | --- | --- | --- |
| **Number of subjects** | **33** | **33** |  |
| Age | 66.72 (15.00) | 66.72 (13.83) | <0.001 |
| Body mass index | 27.50 (5.58) | 26.76 (6.35) | 0.124 |
| Neurological criteria, n (%) | 1.9 ( 5.8) | 1.7 ( 5.2) | 0.028 |
| Number of respiratory criteria |  |  | 0.022 |
| 0-1 | 12.9 (39.7) | 12.7 (39.0) |  |
| 2-3 | 15.9 (48.8) | 15.9 (48.9) |  |
| 4-6 | 3.7 (11.4) | 3.9 (12.1) |  |
| Pulmonary sepsis, n (%) | 10.4 (32.1) | 10.4 (32.1) | <0.001 |
| Respiratory rate (rpm) | 30.12 (7.02) | 30.12 (7.21) | <0.001 |
| PaO_2_/FiO_2_ | 169.32 [100.49, 309.85] | 186.91 [102.02, 285.30] | <0.001 |
| PaCO_2_ (mmHg) | 32.91 (10.55) | 32.91 (11.03) | <0.001 |
| Accessory muscle use, n (%) | 13.3 (40.7) | 13.3 (40.7) | <0.001 |
| Inability to clear secretions, n (%) | 13.3 (40.7) | 13.3 (40.7) | <0.001 |
| pH | 7.31 (0.11) | 7.31 (0.09) | <0.001 |
| Lactate (mmol/L) | 4.16 [2.60, 5.20] | 3.20 [2.20, 5.60] | <0.001 |
| Non-respiratory SOFA | 9.00 [8.00, 11.00] | 9.00 [8.00, 11.00] | <0.001 |
| Norepinephrine dose (mcg/kg/min) | 0.50 [0.27, 0.80] | 0.55 [0.29, 0.80] | <0.001 |
| Glasgow Coma Scale | 13.00 [12.00, 13.00] | 13.00 [12.00, 13.00] | <0.001 |
| Platelet count, (10^12/L) | 153.52 (129.94) | 153.52 (119.02) | <0.001 |
| Bilirubin, (mmol/L) | 19.00 [9.29, 35.15] | 18.00 [12.00, 36.79] | 0.203 |
| Creatinine, (mmol/L) | 217.30 (143.30) | 217.30 (180.12) | <0.001 |
| Renal replacement at T8 | 1.3 ( 3.9) | 1.3 ( 3.9) | <0.001 |
| Median days of MV by center | 3.00 [3.00, 6.00] | 5.00 [3.00, 6.00] | <0.001 |
| Intubation rate by center (%) | 0.21 [0.18, 0.32] | 0.24 [0.17, 0.32] | 0.018 |
| Mortality rate by center (%) | 0.23 [0.20, 0.27] | 0.23 [0.20, 0.29] | <0.001 |

### Table S8: Matched population when comparing early and late intubation using an 8-hour cutoff. Continuous variables are expressed as mean and standard deviation or median and interquartile range.

| **Early intubation** | **Yes** | **No** | **SMD** |
| --- | --- | --- | --- |
| **Number of subjects** | **88** | **88** |  |
| Age | 63.67 (16.16) | 66.77 (11.38) | 0.222 |
| Body mass index | 26.83 (6.20) | 27.89 (7.35) | 0.156 |
| Neurological criteria, n (%) | 6 ( 6.8) | 4 ( 4.5) | 0.098 |
| Number of respiratory criteria |  |  | 0.315 |
| 0-1 | 36 (40.9) | 33 (37.5) |  |
| 2-3 | 36 (40.9) | 47 (53.4) |  |
| 4-6 | 16 (18.2) | 8 (9.1) |  |
| Pulmonary sepsis, n (%) | 34 (38.6) | 28 (31.8) | 0.143 |
| Respiratory rate (rpm) | 29.53 (7.30) | 29.18 (6.95) | 0.049 |
| PaO_2_/FiO_2_ | 132.96 [86.00, 232.14] | 155.79 [97.82, 232.14] | 0.011 |
| PaCO_2_ (mmHg) | 32.86 (9.44) | 34.08 (10.10) | 0.124 |
| Accessory muscle use, n (%) | 43 (48.9) | 39 (44.3) | 0.091 |
| Inability to clear secretions, n (%) | 26 (29.5) | 15 (17.0) | 0.299 |
| pH | 7.32 (0.12) | 7.30 (0.08) | 0.162 |
| Lactate (mmol/L) | 2.90 [2.30, 4.70] | 3.70 [2.28, 5.32] | 0.063 |
| Non-respiratory SOFA | 9.00 [7.00, 11.00] | 8.00 [7.00, 9.00] | 0.176 |
| Norepinephrine dose (mcg/kg/min) | 0.50 [0.30, 0.80] | 0.47 [0.20, 0.76] | 0.147 |
| Glasgow Coma Scale | 15.00 [14.00, 15.00] | 15.00 [14.00, 15.00] | 0.123 |
| Platelet count, (10^12/L) | 128.03 (105.67) | 147.14 (108.55) | 0.178 |
| Bilirubin, (mmol/L) | 16.00 [10.00, 32.00] | 16.00 [12.00, 34.25] | 0.042 |
| Creatinine, (mmol/L) | 194.56 (123.36) | 191.39 (147.16) | 0.023 |
| Renal replacement at T8 | 5 ( 5.7) | 3 ( 3.4) | 0.109 |
| Median days of MV by center | 4.00 [3.00, 6.62] | 6.00 [3.00, 6.12] | 0.167 |
| Intubation rate by center (%) | 0.21 [0.17, 0.32] | 0.22 [0.18, 0.32] | 0.109 |
| Mortality rate by center (%) | 0.23 [0.20, 0.29] | 0.23 [0.21, 0.27] | 0.167 |

### Table S9: Weighted population (using overlap weighting) when comparing early and late intubation using an 8-hour cutoff. Continuous variables are expressed as mean and standard deviation or median and interquartile range.

| **Early intubation** | **Yes** | **No** | **SMD** |
| --- | --- | --- | --- |
| **Number of subjects** | **32** | **32** |  |
| Age | 66.12 (15.35) | 66.12 (10.89) | <0.001 |
| Body mass index | 27.14 (5.89) | 27.82 (7.33) | 0.102 |
| Neurological criteria, n (%) | 1.6 ( 5.0) | 1.5 ( 4.7) | 0.014 |
| Number of respiratory criteria |  |  | 0.044 |
| 0-1 | 14.0 (43.4) | 13.6 (42.2) |  |
| 2-3 | 14.1 (43.8) | 14.8 (45.9) |  |
| 4-6 | 4.1 (12.7) | 3.8 (11.9) |  |
| Pulmonary sepsis, n (%) | 10.5 (32.5) | 10.5 (32.5) | <0.001 |
| Respiratory rate (rpm) | 29.55 (6.85) | 29.55 (6.86) | <0.001 |
| PaO_2_/FiO_2_ | 152.42 [90.67, 266.36] | 154.74 [92.74, 236.66] | <0.001 |
| PaCO_2_ (mmHg) | 33.72 (10.55) | 33.72 (10.08) | <0.001 |
| Accessory muscle use, n (%) | 13.9 (43.0) | 13.9 (43.0) | <0.001 |
| Inability to clear secretions, n (%) | 6.5 (20.3) | 6.5 (20.3) | <0.001 |
| pH | 7.31 (0.11) | 7.31 (0.09) | <0.001 |
| Lactate (mmol/L) | 3.08 [2.28, 4.80] | 3.50 [2.20, 5.30] | <0.001 |
| Non-respiratory SOFA | 8.77 [7.00, 11.00] | 8.00 [7.00, 10.00] | <0.001 |
| Norepinephrine dose (mcg/kg/min) | 0.50 [0.27, 0.80] | 0.48 [0.25, 0.89] | <0.001 |
| Glasgow Coma Scale | 15.00 [14.00, 15.00] | 15.00 [14.00, 15.00] | <0.001 |
| Platelet count, (10^12/L) | 134.77 (105.99) | 134.77 (98.81) | <0.001 |
| Bilirubin, (mmol/L) | 17.92 [10.83, 32.00] | 15.00 [11.00, 35.01] | 0.153 |
| Creatinine, (mmol/L) | 197.93 (136.77) | 197.93 (139.51) | <0.001 |
| Renal replacement at T8 | 1.2 (3.7) | 1.2 (3.7) | <0.001 |
| Median days of MV by center | 4.42 [3.00, 7.00] | 5.20 [3.00, 6.00] | <0.001 |
| Intubation rate by center (%) | 0.20 [0.18, 0.32] | 0.22 [0.17, 0.32] | 0.022 |
| Mortality rate by center (%) | 0.23 [0.20, 0.27] | 0.23 [0.20, 0.27] | <0.001 |

### Table S10: Outcomes in the sensitivity analyses. Early intubated subjects vs unexposed individuals. *Number of subjects intubated after 8 hours (in the first 2 analyses) or after 24 hours (in the third analysis). | Unexposed refers to subjects not having received intubation in the first 8 hours (or 24 hours in the third analysis). ICU and Hospital length of stay, up to 60 days after ICU admission, and organ dysfunction parameters at 24 hours. First value shown represents mean (or median) in the treated group, second value shows the mean in the control subjects. 95% confidence intervals for the differences are depicted within parentheses.

| **Outcome** | **Overlap weighting** | **Criteria for intubation only** | **Exposure up to 24h** | **Complete case only** | **Early vs late intubation with matching** | **Early vs late intubation with weighting** |
| --- | --- | --- | --- | --- | --- | --- |
| Number (%) of early-intubated subjects included | 54 (39%) | 29 (32%) | 67 (36%) | 33 (39%) | 88 (64%) | 32 (23%) |
| Mortality, number (%) | 22 (40) vs 15 (27), p= 0.22 | 14 (48) vs 9 (30),  p= 0.28 | 29 (43) vs 19 (27),  p= 0.07 | 15 (45) vs 9 (27),  p= 0.21 | 37 (42) vs 39 (44), p= 0.88 | 13 (41) vs 14 (42), p= 1 |
| Hazard ratio | 1.60 (1.009-2.55),  p= 0.045 | 1.87 (1.04-3.37),  p= 0.04 | 1.69 (1.10-2.61), p= 0.01 | 1.88 (1.06-3.34),  p= 0.03 | 1.02 (0.65-1.90),  p= 0.92 | 1.06 (0.63-1.80), p= 0.80 |
| Late intubation among unexposed, number (%)\| | 18 (34%) | 12 (43%) | 11 (16%) | 16 (33%) | 88 (100%) | 32 (100%) |
| Median day of intubation among late-intubated subjects | 2 [1-3] | 1 [1-2] | 2 [1-3] | 1 [1-2] | 1 [1-2] | 1 [1-3] |
| Treatment limitation orders, number (%) | 14 (26) vs 12 (12), p= 0.61 | 8 (27) vs 7 (26),  p= 0.88 | 19 (28) vs 19 (28),  p= 0.98 | 9 (29) vs 8 (25),  p= 0.61 | 26 (30) vs 23 (26), p= 0.74 | 9 (27) vs 9 (27), p= >0.99 |
| ICU length of stay (days) | 7 vs 4 (1 to 6) | 7 vs 5 (0 to 7) | 9 vs 4 (1-7) | 9 vs 6 (0 to 7) | 9 vs 10 (-7 to 3) | 9 vs 10 (-9 to 3) |
| Hospital length of stay (days) | 12 vs 13 (-8 to 5) | 10 vs 19 (-12 to 7) | 16 vs 16 (-6 to 6) | 18 vs 20 (-11 to 5) | 17 vs 22 (-15 to 4) | 18 vs 22 (-15 to 1) |
| Non-respiratory, non-neurologic SOFA score | 8 vs 7 (0 to 2) | 8 vs 7 (-1 to 2) | 7 vs 7 (0 to 2) | 7 vs 7 (-1 to 2) | - | - |
| Noradrenaline (mcg/kg/min) | 0.65 vs 0.21 (0.19 to 0.70) | 0.69 vs 0.29 (0.12 to 0.70) | 0.69 vs 0.10 (0.35 to 0.80) | 0.67 vs 0.29 (0.11-0.83) | - | - |
| Lactate (mmol/L) | 2.3 vs 2.1 (-0.2 to 0.9) | 3.0 vs 2.0 (0.10 to 2.3) | 2.5 vs 1.9 (0.05-1.4) | 2.2 vs 1.9 (-0.4 to 0.8) | - | - |
| Fluid balance (mL) | 2700 vs 1100 (800-2300) | 3400 vs 1300 (800-3100) | 2700 vs 900 (1200-2400) | 2800 vs 1200 (800-2500) | - | - |
| Renal replacement | 35% vs 14% (5-37) | 33 vs 14% (2-36) | 53 vs 13% (13-67) | 34 vs 14% (3-36) | - | - |

**FIGURES**


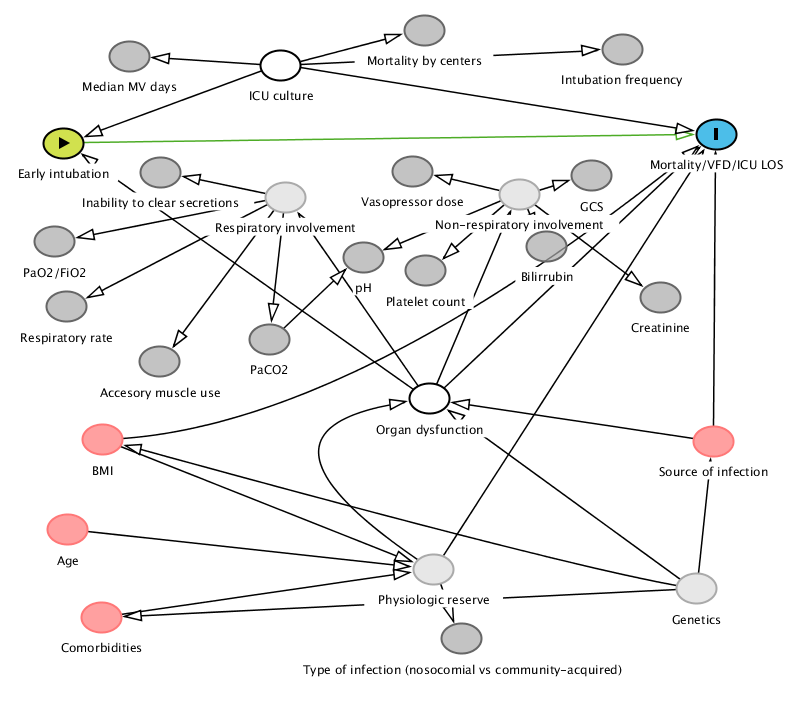


### *Figure S1: Direct acyclic graph (DAG) for the assessment of early intubation on mortality, ventilator-free days (VFD) and ICU length of stay. In green, on the upper left top, the exposure of interest. In blue, on the upper right top, the outcome of interest. Confounders, which are variables linked both to the exposure and the treatment, are depicted in red (observed), white (adjusted by surrogates) or light grey (unmeasured). Surrogate variables (in dark grey) were used to estimate organ dysfunction. Given the structure of this DAG, only adjustment for the degree of organ dysfunction and ICU culture was required in order to estimate the causal effect of early intubation on mortality, since the additional measured and unmeasured confounders were also linked to the degree of organ dysfunction.*

*
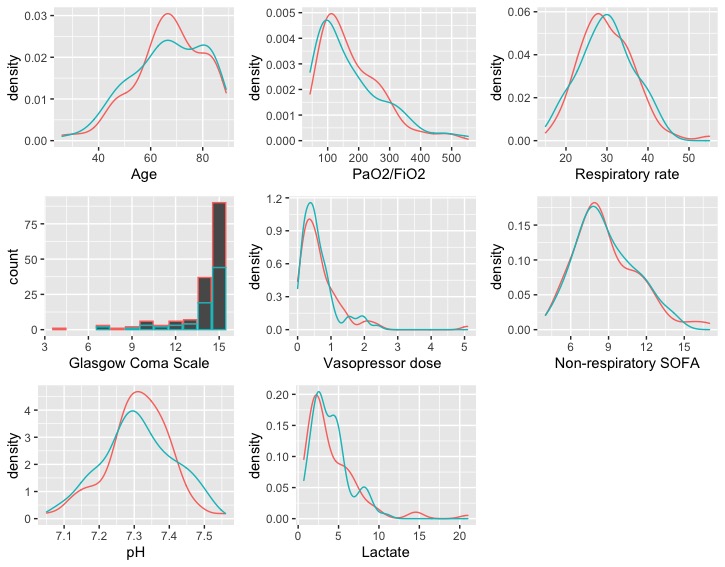
*

### *Figure S2: Distribution of covariates in the matched population. Red denotes unexposed subjects while green denotes early intubated individuals.*

*
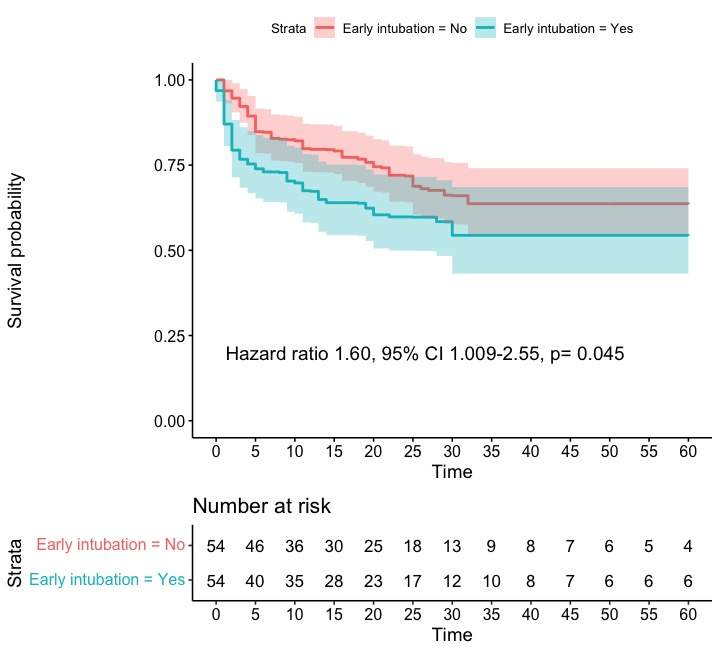
*

### *Figure S3: Kaplan Meier curves when the adjusted population was created with overlap weighting.*

*
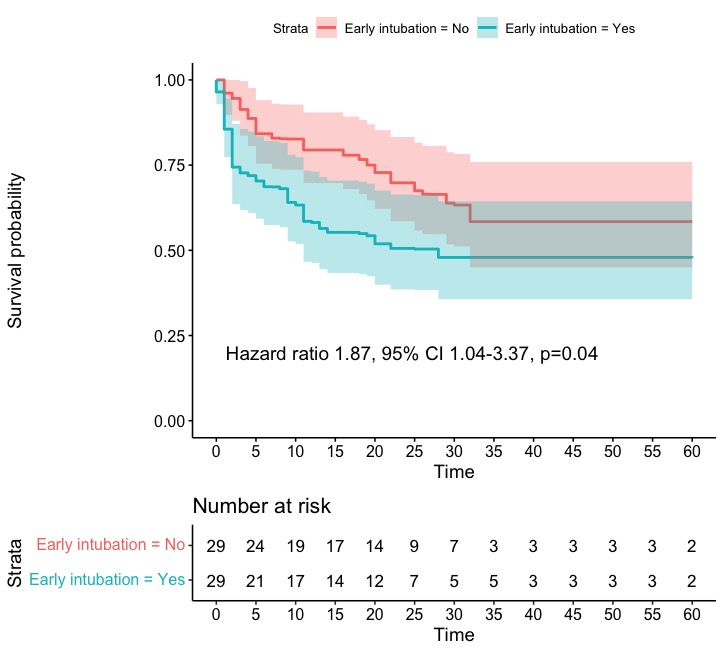
*

### *Figure S4: Kaplan Meier curves for the comparison of patients displaying immediate criteria for intubation.*


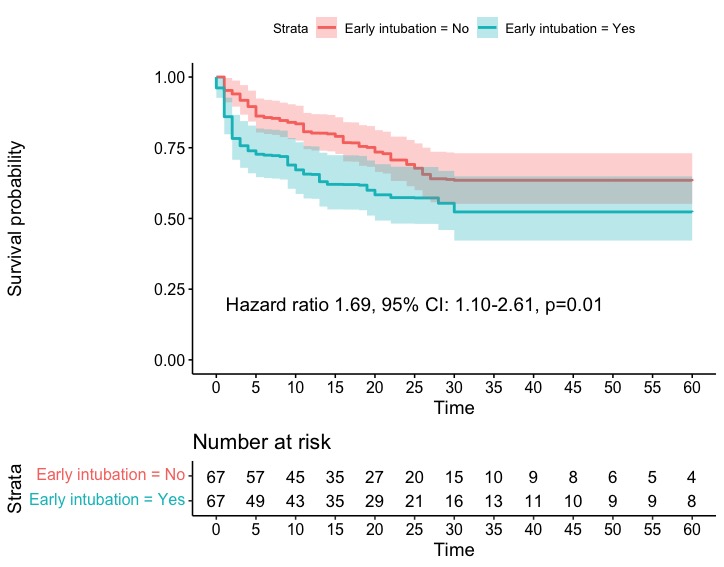


### *Figure S4: Kaplan Meier curves for the comparison of patients after modifying exposure up to 24 hours.*


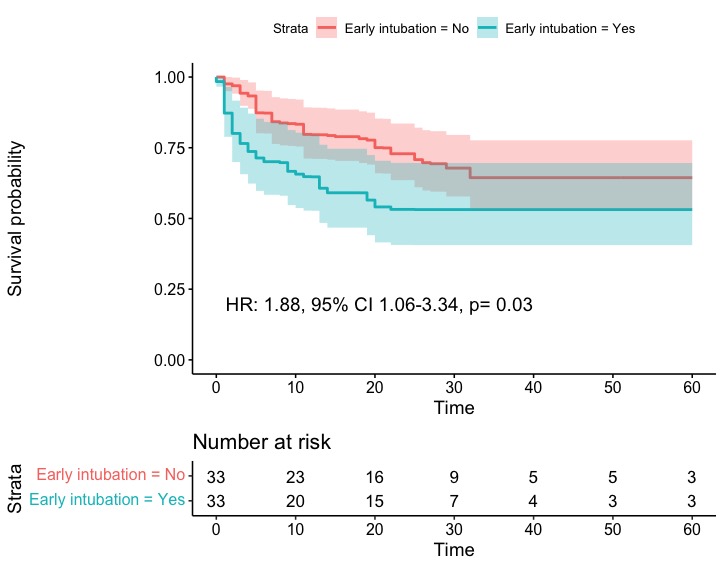


### *Figure S5: Kaplan Meier curves for the comparison of patients after carrying out a complete-case analysis.*


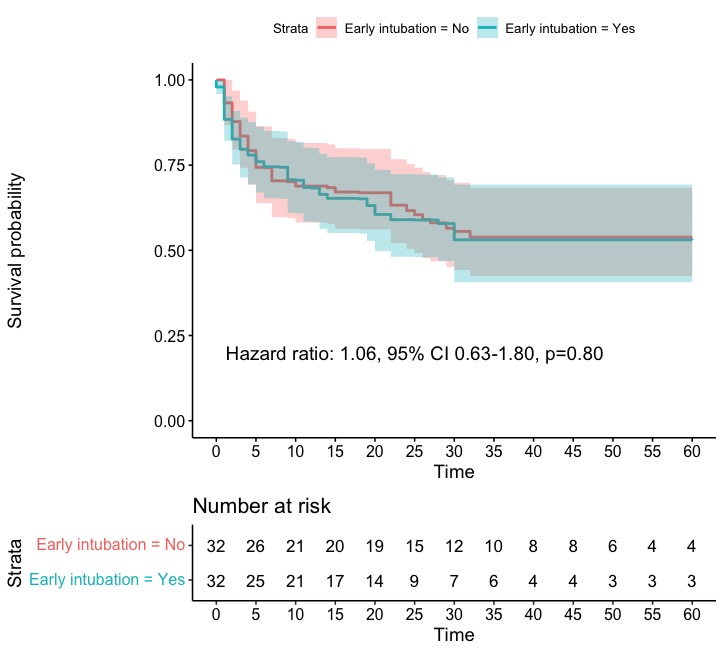


### *Figure S6: Comparison between patients who were intubated early or late (beyond 8 hours) using overlap weighting.*

## **REFERENCES:**

1. Etminan M, Collins GS, Mansournia MA. Using Causal Diagrams to Improve the Design and Interpretation of Medical Research. *Chest*. 2020;158(1):S21-S28. doi:10.1016/j.chest.2020.03.011

2. van Buuren S, Groothuis-Oudshoorn K. mice: Multivariate imputation by chained equations in R. *J Stat Softw*. 2011;45(3):1-67. doi:10.18637/jss.v045.i03

3. Zhang Z. Multiple imputation with multivariate imputation by chained equation (MICE) package. *Ann Transl Med*. 2016;4(2):30. doi:10.3978/j.issn.2305-5839.2015.12.63

4. Austin PC. A tutorial and case study in propensity score analysis: An application to estimating the effect of in-hospital smoking cessation counseling on mortality. *Multivariate Behav Res*. 2011;46(1):119-151. doi:10.1080/00273171.2011.540480

5. Desai RJ, Franklin JM. Alternative approaches for confounding adjustment in observational studies using weighting based on the propensity score: A primer for practitioners. *BMJ*. 2019;367. doi:10.1136/bmj.l5657

6. Austin PC, Small DS. The use of bootstrapping when using propensity-score matching without replacement: A simulation study. *Stat Med*. 2014;33(24):4306-4319. doi:10.1002/sim.6276
